# Big Data-Driven Determinants of Length of Stay for Patients with Hip Fracture

**DOI:** 10.3390/ijerph17144949

**Published:** 2020-07-09

**Authors:** Jihye Lim

**Affiliations:** Department of Healthcare Management, Youngsan University, Yangsan 50510, Korea; limjiart@ysu.ac.kr

**Keywords:** length of stay (LOS), hip fractures, comorbidity

## Abstract

It is important that length of stay (LOS) management for alleviating health care financial burdens and improving patient outcomes. The aim of this study was to report the differences of LOS and the factors affecting LOS of hip fracture patients using big data. A total of 463,194 data were collected from 2016 to 2017 KNHDS. Of those, 2238 patients with the hip fracture primary diagnosis were included in the study population. As independent variables were used gender, age, type of insurance, admission route, result of treatment, number of hospital beds, the presence of surgery, and comorbidities. Statistical analysis performed using the IBM SPSS Statistics for Windows, version 25.0. A statistically significant difference was observed in the length of stay of hip fracture patients according to the healthcare insurance type. The difference in LOS associated with comorbidities was statistically significant for hypertension, peptic ulcer disease, coagulopathy, and alcohol abuse (*p* < 0.05). Independent variables that affected LOS of hip fracture patients with national health insurance were the treatment result, operation presence, comorbidity count, and hospital beds (*p* < 0.001). The factors associated with the length of stay for hip fracture patients were the difference according to the healthcare insurance type. The results of this study can be used as a basic data for the national health policy for the proper distribution and utilization of medical resources.

## 1. Introduction

Hip fracture is one of the major diseases requiring orthopedic hospitalization. It is usually caused by falls in older people with osteoporosis or osteopenia [1]. Hip fractures account for 63–72% of all fracture admissions in patients over 50 years of age and continue to occur significant costs for one year after the fracture [2,3]. Hip fractures cause many patients to lose mobility and independence and affect a major impact on health-related quality of life. Patients with femur fractures are known to have a high risk of premature death and a high mortality rate [4,5,6]. However, most of these deaths are not due to the hip fracture itself, but to the associated other conditions and comorbidities that predominantly affect elderly patients [1].

The hospital treatment of hip fractures is a complex process that involves multiple services [7,8]. The length of stay, mortality, and other health outcome indicators are different according to the treatment process that includes various factors. The National Institute for Health and Care Excellence (NICE) guidelines for treatment of femoral fracture include a number of standards: prompt admission to orthopedic care; surgery within 36 h and within normal working hours; nursing care aimed at minimizing pressure ulcer incidence; routine access to ortho-geriatric medical care; assessment and appropriate treatment to promote bone health; and falls assessment [9].

According to previous studies, it is known that there are differences in the length of stays for injured patients depending on the type of payer and socio-economic status [10]. Yoon and Kwon et al. reported that fracture patients with workers’ compensation insurance had a longer length of stay than patients with national health insurance [11,12]. In general, 20% of the total length of stay represents unnecessary hospital days in patients with workers’ compensation insurance [10]. These unnecessary long-term hospitalization implying an important waste of resources as well as increased patients’ exposure to adverse events and weaken physical function [13,14,15]. The reduction in the length of stay has been identified as one of the key strategies for alleviating health care financial burdens and improving patient outcomes [16].

Despite the social burden of the hip fracture, research on the factors that affect its costs and length of stay is scarce. It is necessary to present it as a material for the appropriateness and effectiveness of the use of medical services through a study of the differences in the length of stays by the types of insurance and the causes of prolonged length of stays.

The purpose of this study is to grasp the differences of the length of stay depending on the type of insurance for hip fracture patients, and the factors affecting the length of stay. It will suggest essential data for the establishment of the health care policy for improvement of the quality of medical care.

## 2. Subjects and Methods

The data used in this study was taken from the Korean National Hospital Discharge In-depth Injury Survey (KNHDS), performed from 2016 to 2017 by Korea Centers for Disease Control and Prevention (KCDC). The KNHDS is collected data from about 150 hospitals with more than 100 beds nationwide and the survey items include gender, age, type of insurance, primary diagnosis, secondary diagnosis, admission route, length of stay, discharge type, treatment results, etc. A total of 463,194 data were collected from 2016 to 2017 KNHDS. Of those, 2238 patients with a hip fracture primary diagnosis were included in the study population.

Hip fracture was defined using any of the following International Classification of Diseases, 10th revision (ICD-10) diagnosis: femoral neck fracture (ICD-10 code S72.0), pertrochanteric fracture (ICD-10 code S72.1), or subtrochanteric fracture (ICD-10 code S72.2). 

The surgical procedures included were total or partial prosthetic replacement, open or closed reduction, and internal fixation of the hip joint. The dependent variable was the length of stay of hip fracture patients. As independent variables showing social demographic characteristics, we used gender, age, and type of insurance. Type of insurance was defined as national health insurance, Medicare, and others (the workers’ compensation insurance, car accident insurance, etc.). The admission route, result of treatment, number of hospital beds, and the presence of surgery were used as independent variables showing medical use characteristics. The result of treatment was divided into improved and not improved. Variables indicating the severity of the fracture were the accompanying comorbidities. We identify comorbidities for each patients, by using the presence of disease that were described in the Elixhauser comorbidity index. The Elixhauser comorbidity measure included 31 conditions, is known to be a better predictor of mortality in patients with cardiac, gastrointestinal, hepatobiliary, and oncologic conditions. The Elixhauser measure included several prevalent comorbidities such as hypertension, obesity, weight loss, and psychiatric disorders [17].

Statistical analysis performed using the IBM SPSS Statistics for Windows, version 25.0 (IBM, Armonk, NY, USA). ANOVA and *t*-test analyses were conducted to ascertain the difference of length of stay according to social demographic characteristics, comorbidities, and medical use characteristics of hip fracture patients. Multiple regression analysis was conducted to determine factors that affect the length of stay of patients with a hip fracture according to the insurance type. In November 2019, to acquire the data for the present study, the researchers went through the procedure for consent, including the application form for the use of raw materials and the pledge of information security through the injury Monitoring Business Homepage of Center for Disease Control and Prevention (Statics Korea, approval No. 117060). The KNHDS data is regarded as accurate data because it is a nationally approved statistic provided by the Korea Centers for Disease Control and Prevention.

## 3. Results

Descriptive statistics for patient characteristics, comorbidities, and medical use characteristics variables according to the insurance type are presented in Table 1. In the study population, most patients were female (68.8%) and 65 years old and older (83.6%). Of the patients 82.7% were admitted into hospital through the emergency room and 91.9% of patients were discharged with improved treatment results. The rate of hip fracture surgery was 81.0% and hospital use by the hospital bed size was the highest 42.6% at 500–999 beds followed by 39.2% at 100–299 beds (Table 1).

**Figure 1:**
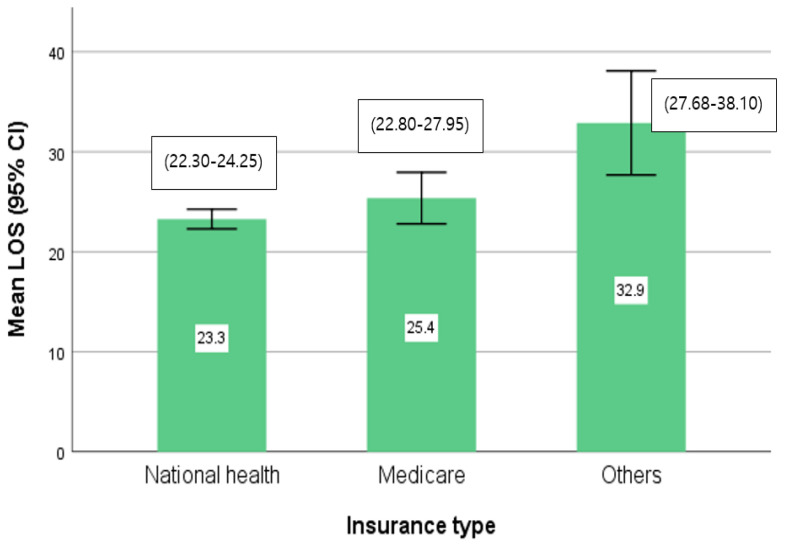
Mean length of stay (LOS) according to insurance type.

Results of the mean LOS according to insurance type were highest at 32.9 days at the other types of insurance, including workers’ compensation and car insurance, followed by 25.4 days at Medicare insurance and 23.3 days at the national health insurance (Figure 1).

Table 2 shows the prevalence of comorbidities and difference of LOS according to the Elixhauser comorbidity index in our study population. The variable of comorbidity means the presence or absence of each disease. Patients without hypertension are not hypertension, but other comorbidities (congestive heart failure, renal failure, diabetes, etc.) can be included in some cases. Hypertension was the main comorbidity with a prevalence of 26.5%, followed by diabetes (15.4%) and renal failure (4.0%). The difference in LOS associated with comorbidities was statistically significant for hypertension, peptic ulcer disease, coagulopathy, and alcohol abuse (*p* < 0.05; Table 2). 

LOS according to the general characteristics of national health insurance patients were statistically significant different in the treatment result, operation presence, and hospital beds (*p* < 0.01). In the cases where the treatment result was ‘improved’, LOS (24.5 days) was longer than those ‘not improved’ (9.89 days), and longer when surgery was performed (24.83 days) than when it is not performed (16.43 days). In the case of 300–499 hospital beds, LOS was the longest than other hospital beds. LOS according to the general characteristics of Medicare patients were statistically significant different in the operation presence, and hospital beds (*p* < 0.05). LOS according to the general characteristics of other insurances (workers’ compensation, car insurance, etc.) were statistically significant different in age, treatment result, and hospital beds (*p* < 0.05; Table 3).

We conducted a multiple regression analysis to identify factors associated with the LOS of hip fracture patients. The independent variables, which significantly affected the LOS of hip fracture patients with national health insurance were the treatment result, operation presence, comorbidity count, and hospital beds (*p* < 0.001). It can be seen that the LOS of hip fracture patients significantly increased, according to the number of comorbidities increase and if the operation was performed. However, if the treatment result had ‘ not improved’, LOS significantly decreased. LOS of hip fracture patients in which the hospital beds had 500 or more beds was significantly lower than in the case that hospital beds had 100–299 beds. The adjusted R-squared was 0.84 and the F statistics value was 19.839 (*p* < 0.001), it can be seen that regression model was a statistically significant model. The Durbin–Watson statistic value was 1.864, which is relatively close to 2, indicating that there was no problem of independence and autocorrelation of the error term. The independent variables, which significantly affected LOS of hip fracture patients with Medicare insurance were age, operation presence, comorbidity count, and hospital beds (*p* < 0.05). The independent variables that significantly affected LOS of hip fracture patients with other insurances (workers’ compensation, car insurance, etc.) were the treatment result and hospital beds (*p* < 0.05; Table 4).

## 4. Discussion

This study was designed to examine factors associated with LOS for hip fracture patients according to the healthcare insurance type, using the nationwide KNHDS data. In this study, there was a significant difference in the LOS of hip fracture patients according to the healthcare insurance type and comorbidities. This result is consistent with a previous study that showed that LOS of the workers’ compensation insurance hip fracture patients was 2.4 times longer than that of the national health insurance patients [11,18]. It can be considered that the difference in the length of stay according to the insurance type is the result of not only the doctor’s judgment on the treatment process but also the influence of patients who want to be more compensated [19]. Additionally, consistent with Nikkel et al. [20], we found that comorbidities such as hypertension, peptic ulcer disease, coagulopathy, and alcohol abuse were important determinants of LOS for hip fracture. We also found that if the treatment result was ‘improved’ and the operation was performed, LOS in all type insurance patients was higher. This result is consistent with a previous study [11] and associated with the study result that showed that causes for differences in LOS was most likely because of different surgical methods [21]. On the other hand, in the case of health insurance and Medicare, there was no significant difference in the length of stay among age groups, but for other insurance (workers’ compensation, car insurance, etc.), the length of stay for the 45–64 age group increased. This suggests not only that the occupational accident rate of older workers is relatively higher than that of younger workers, but also that injury severity is higher [22]. In addition, in the case of health insurance and Medicare, the length of stay was significantly higher when surgery was performed than when it was not, but in the case of other insurance (workers’ compensation, car insurance, etc.), where it was shown to have no significant differences. These results also suggest that in the case of occupational accidents and car insurance, the length of stay required for financial compensation tended to be long, regardless of the course of treatment, because of the benefit of patients and medical institutions.

According to the results of this study, the percentage of those who did not undergo surgery was 19%. As for the treatment of hip joint disease, the major principle was to perform surgical treatment. Even with the development of medicine, mortality is still reported to be 15–30% within 1 year after hip fracture [23]. A clinical problem is that most patients with hip fractures have one or more medical diseases. In fact, according to a previous study of hip fractures in South Korea, it was found that elderly patients aged 85 years or older and when their BMI was low and when their physical condition difficult to anesthesia chose non-surgical treatment [24]. Osteoporosis is also known to increase the risk of developing hip joint disease, and the incidence of osteoporosis in people over the age of 50 in South Korea increased by an average of 15.2% per year [25] The risk of hip fractures in the population over the age of 50 in South Korea was investigated in 5.3% of men and 12.3% of women, and the risk of hip fracture in Japan was investigated in 5.6% of men and 20.0% of women [26,27].

For the multiple regression analysis result, the factors associated with the length of stay for hip fracture patients were the treatment result, operation presence, comorbidity count, hospital bed number, etc., and these associated factors were different according to the healthcare insurance type. This finding implies that different health policies and approaches should be considered for each healthcare insurance type by a population-based method.

Administrative data such Korean National Hospital Discharge In-depth Injury Survey (KNHDS) data have intrinsic strengths and weakness for studies of this nature [28]. The strength of the analytical data is that it is representative big data, relatively accurate data that has been refined by experts. The principal weakness of this study include a cross-sectional study design that was used, thus it was not possible to establish causal relationships between LOS and other variables. Another limitation is the lack of clinical information on aspects known to affect LOS, such as information regarding disease severity, post-operative complication, and surgical procedure type. Despite these limitations, this work analyzed nationwide data by adding the types of healthcare insurance and comorbidity variables that could affect the LOS. In the future, studies that include medical expenses of hip fracture diseases and LOS relevant variables should be conducted. This study suggests the establishment of the health care policy according to the target population based on an accurate analysis, in order to improve the quality and efficiency of medical care. Furthermore, the results of this study can be used as a basic data for the national health policy for the proper distribution and utilization of medical resources.

## 5. Conclusions

Results suggest that factors associated with the length of stay for hip fracture patients were difference according to the healthcare insurance type. This suggests that some unnecessary length of stay is being induced depending on the type of medical insurance, and it seems that appropriate distribution and utilization of medical resources are required. Therefore, it is considered necessary to conduct a bed utilization review based on the medical need at the hospitalization stage in the medical institution to mitigate the unnecessary extension of the length of stay. In particular, for diseases with a length of stay, it is necessary to ensure patient safety and efficient utilization of medical resources through appropriate interventions of the case manager in three stages before hospitalization, during hospitalization, and after discharge.

## Figures and Tables

**Table 1:** Description of the study population according to the insurance type.

Variables	National Health	Medicare	Others	Total
	*N* (%)	*N* (%)	*N* (%)	*N* (%)
Gender				
Male	528 (28.6)	72 (27.9)	99 (72.8)	699 (31.2)
Female	1316 (71.4)	186 (72.1)	37 (27.2)	1539 (68.8)
Age				
≤44	57 (3.1)	6 (2.3)	27 (19.9)	90 (4.0)
45–64	188 (10.2)	42 (16.3)	47 (34.6)	277 (12.4)
≥65	1599 (86.7)	210 (81.4)	62 (45.6)	1871 (83.6)
Admission route				
Emergency	1525 (82.7)	211 (81.8)	115 (84.6)	1851 (82.7)
Outpatient	319 (17.3)	47 (18.2)	21 (15.4)	387 (17.3)
Result of treatment				
Improved	1690 (91.6)	236 (91.5)	130 (95.6)	2056 (91.9)
Not improved	154 (8.4)	22 (8.5)	6 (4.4)	182 (8.1)
Operation				
No	341 (18.5)	53 (20.5)	31 (22.8)	425 (19.0)
Yes	1503 (81.5)	205 (79.5)	105 (77.2)	1813 (81.0)
Hospital beds				
100–299	694 (37.6)	131 (50.8)	52 (38.2)	877 (39.2)
300–499	160 (8.7)	28 (10.9)	19 (14.0)	207 (9.2)
500–999	815 (44.2)	85 (32.9)	54 (39.7)	954 (42.6)
≥1000	175 (9.5)	14 (5.4)	11 (8.1)	200 (8.9)

**Table 2:** Difference of the length of stay according to the Elixhauser comorbidity index.

Comorbidity	Number of Patients, (%)	Mean LOS, SD		*t*	*p*
		Yes	No		
Congestive heart failure	41 (1.8)	26.63 ± 21.04	24.05 ± 22.17	−0.74	0.46
Cardiac arrhythmia	60 (2.7)	23.12 ± 18.28	24.13 ± 22.25	0.35	0.73
Valvular disease	24 (1.1)	24.50 ± 20.30	24.10 ± 22.18	−0.09	0.93
Pulmonary circulation disease	20 (0.9)	23.55 ± 13.94	24.11 ± 22.21	0.11	0.91
Peripheral vascular disease	18 (0.8)	29.61 ± 20.89	24.06 ± 22.16	−1.06	0.29
Hypertension	594 (26.5)	25.64 ± 23.60	23.55 ± 21.59	−1.97	<0.05
Hypertension, complicated	13 (0.6)	26.69 ± 19.77	24.09 ± 22.17	−0.42	0.67
Paralysis	9 (0.4)	45.00 ± 44.14	24.02 ± 21.20	−1.43	0.19
Neurological disorder	46 (2.1)	27.41 ± 20.43	24.03 ± 22.19	−1.03	0.31
Chronic pulmonary disease	60 (2.7)	28.83 ± 20.13	23.97 ± 22.20	−1.68	0.94
Diabetes	345 (15.4)	25.70 ± 25.77	23.81 ± 21.42	−1.46	0.15
Diabetes, complicated	38 (1.7)	30.92 ± 18.29	23.98 ± 22.19	−1.92	0.06
Hypothyroidism	20 (0.9)	27.90 ± 22.48	24.07 ± 22.15	−0.77	0.44
Renal failure	90 (4.0)	25.04 ± 19.53	24.06 ± 22.26	−0.41	0.68
Liver disease	30 (1.3)	30.80 ± 21.70	24.01 ± 22.15	−1.67	0.10
Peptic ulcer disease	6 (0.3)	45.83 ± 19.22	24.04 ± 22.14	−2.41	<0.05
Lymphoma	2 (0.1)	13.00 ± 5.66	24.11 ± 22.16	0.71	0.48
Metastatic cancer	4 (0.2)	26.00 ± 14.17	24.10 ± 22.17	−0.17	0.86
Solid tumor	19 (0.8)	34.00 ± 29.67	24.02 ± 22.07	−1.46	0.16
Rheumatoid arthritis	9 (0.4)	21.33 ± 13.71	24.11 ± 22.18	0.38	0.71
Coagulopathy	9 (0.4)	40.89 ± 26.72	24.03 ± 22.11	−2.28	<0.05
Weight loss	3 (0.1)	21.67 ± 0.58	24.10 ± 22.17	0.19	0.85
Electrolyte disorder	34 (1.5)	29.53 ± 23.80	24.02 ± 22.12	−1.44	0.15
Deficiency anemia	22 (1.0)	27.91 ± 25.45	24.06 ± 22.12	−0.81	0.42
Alcohol abuse	12 (0.5)	40.75 ± 26.84	24.01 ± 22.10	−2.61	<0.05
Psychosis	13 (0.6)	48.00 ± 57.47	23.96 ± 21.74	−1.51	0.16
Depression	32 (1.4)	34.13 ± 58.19	23.96 ± 21.18	−0.99	0.33

If less than 2 cases of disease is excluded from the analysis. SD: Standard Deviation.

**Table 3:** Difference of the length of stay according to general characteristics.

Variables	National Health		Medicare		Others	
	Mean, SD	*p*	Mean, SD	*p*	Mean, SD	*p*
Gender						
Male	23.93 ± 25.27	0.45	24.85 ± 16.36	0.80	35.08 ± 33.92	0.18
Female	23.01 ± 19.65		25.58 ± 22.60		27.03 ± 18.97	
Age						
≤44	25.95 ± 47.35	0.26	32.33 ± 20.21	0.33	26.41 ± 20.54	<0.05
45–64	21.23 ± 18.73		28.88 ± 31.04		43.77 ± 44.06	
≥65	23.42 ± 20.20		24.47 ± 18.43		27.47 ± 17.48	
Admission route						
Emergency	23.12 ± 19.72	0.49	24.80 ± 18.99	0.35	32.34 ± 26.36	0.63
Outpatient	24.03 ± 28.15		27.96 ± 28.52		35.90 ± 48.99	
Result of treatment						
Improved	24.50 ± 21.54	<0.01	26.08 ± 19.91	0.22	34.30 ± 30.70	<0.05
Not improved	9.89 ± 14.22		17.77 ± 30.05		2.33 ± 3.01	
Operation						
No	16.43 ± 31.90	<0.01	17.53 ± 19.47	<0.01	36.68 ± 51.40	0.61
Yes	24.83 ± 17.86		27.40 ± 20.97		31.77 ± 21.40	
Hospital beds						
100–299	25.67 ± 23.78	<0.01	27.77 ± 23.37	<0.05	38.31 ± 36.63	<0.05
300–499	28.67 ± 21.15		30.46 ± 29.62		45.58 ± 27.32	
500–999	21.39 ± 19.84		20.61 ± 11.09		26.78 ± 25.77	
≥1000	17.66 ± 15.96		21.64 ± 19.35		15.36 ± 6.50	

**Table 4:** Factors associated with the length of stay for hip fracture patients.

Variables	National Health Insurance			Medicare			Others		
	ß	*t*	*p*	ß	*t*	*p*	ß	*t*	*p*
Gender									
Male (ref.)									
Female	−1.896	−1.701	0.089	3.814	1.232	0.219	−7.674	−1.297	0.197
Age	0.018	0.453	0.650	−0.230	−2.220	<0.05	−0.016	−0.111	0.912
Admission route									
Emergency (ref.)									
Outpatient	−0.205	−0.157	0.875	3.136	0.905	0.366	−1.488	−0.206	0.837
Result of treatment									
Improved (ref.)									
Not improved	−13.44	−7.119	<0.001	−2.056	−0.416	0.678	−37.57	−2.876	<0.01
Operation									
No (ref.)									
Yes	6.507	4.766	<0.001	11.276	3.184	<0.01	−9.228	−1.379	0.170
Comorbidity count	2.337	5.019	<0.001	2.642	2.293	<0.05	4.869	1.469	0.144
Hospital beds									
100–299 (ref.)									
300–499	2.089	1.148	0.251	1.223	0.285	0.776	8.559	1.034	0.303
500–999	−6.448	−5.817	<0.001	−9.754	−3.325	<0.01	−12.28	−2.063	<0.05
≥1000	−10.83	−6.117	<0.001	−5.567	−0.969	0.333	−23.73	−2.401	<0.05
Adj. R^2^ F (*p*) Durbin–Watson	0.084 19.839 (0.000) 1.864			0.089 3.798 (0.000) 1.958			0.114 2.924 (0.004) 2.034		
